# Adult Left Colocolic Intussusception Successfully Managed by Left Hemicolectomy and Primary Anastomosis

**DOI:** 10.1055/s-0042-1742751

**Published:** 2022-02-18

**Authors:** Deepak Rajput, Lena Elizabath David, Oshin Sharma, Amit Gupta, Rohik Anjum T. Siddeek, Ravi Hari Phulware

**Affiliations:** 1 Department of General Surgery, All India Institute of Medical Sciences, Rishikesh, Dehradun, Uttarakhand, India; 2 Department of Pathology, All India Institute of Medical Sciences, Rishikesh, Dehradun, Uttarakhand, India

**Keywords:** intussusception, intestinal obstruction, colonic

## Abstract

Intussusception, although quite common in children with the classic triad of cramping abdominal pain, bloody diarrhea, and palpable masses, is a rare cause of acute abdomen with myriad presentations in adults. It is defined as the telescoping of a proximal segment of the gastrointestinal (GI) tract, called the intussusceptum, into the lumen of the adjacent distal segment of the GI tract, called intussuscipiens. Due to its different manifestations and time course, adult colonic intussusception often poses a diagnostic challenge for emergency doctors. The treatment of colonic intussusception in adults typically involves surgery, often with bowel resection and anastomosis followed by a defunctioning loop ileostomy. We report a case of left-sided colocolic intussusception secondary to a tubular adenoma as the lead point, which was successfully treated by resection and primary anastomosis. The pathological diagnosis of the lesion was reported as adenocarcinoma and resected bowel margins were found free of the tumor.

Intussusception represents a rare form of bowel obstruction in adults and is an uncommon cause of abdominal pain, accounting for only 1 to 5% of intestinal obstructions and 5% of all intussusceptions. 1 2 Almost 90% of the cases of intussusception in adults are secondary to a pathologic condition that serves as the lead point, such as carcinoma, polyp, Meckel's diverticulum, colonic diverticulum, stricture, or benign neoplasm, usually discovered intraoperatively. It is extremely rare to see intussusception involving descending colon. Two-thirds of the colonic intussusceptions are associated with primary carcinoma of the colon. Left-sided colon resection and anastomosis are usually done with proximal covering ileostomy unlike right-sided resections. The preferred treatment in such cases is resection of intussusception without initial surgical reduction, to minimize the operative manipulation of potential malignancy.

## Case Report

A 46-year-old man presented to the emergency room with complaints of multiple episodes of loose stools (15–20 episodes/day) mixed with blood and nonprojectile bilious vomiting (10–15 episodes/day) for 2 days. He reports acute-onset severe colicky abdominal pain, associated with abdominal distention during this time period. On general physical examination, a positive finding of pallor was noted. His recorded vitals at presentation were: pulse rate 110 beats/min, blood pressure 110/70 mm Hg, respiratory rate 20 breaths/min, oxygen saturation 98% on room air, and body temperature 98.6°F, and examination of the respiratory, cardiovascular, and central nervous systems revealed no obvious abnormality. His abdominal examination showed gross distension with apparent peristalsis and was diffusely tender with evidence of guarding on palpation. Examination of the external genitalia, hernial orifices, and back revealed no abnormality. On digital rectal examination, rectum was collapsed and the finger was stained with mucus.

**Fig. 1:**
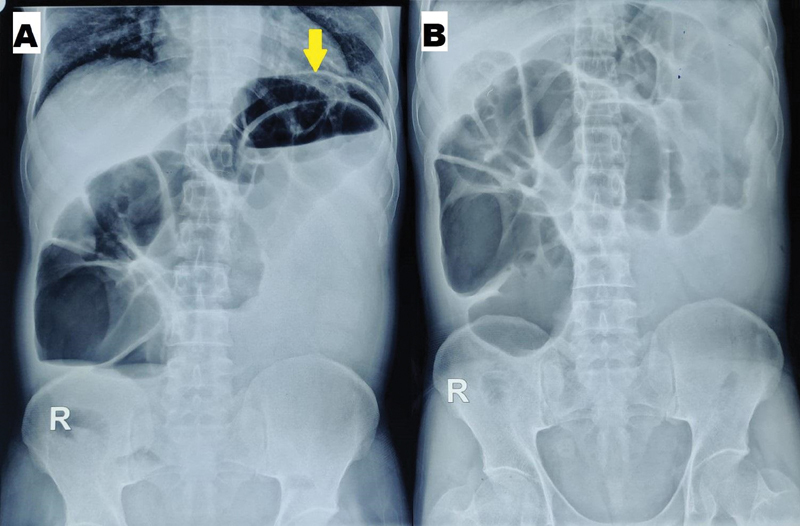
Abdominal radiograph showing ( **A** ) crescent-shaped soft tissue density in an air–fluid level in erect view and ( **B** ) grossly dilated large bowel loops with absent rectal gas shadow in a supine film.

Blood investigations, that included a complete blood count with liver and kidney function tests, revealed values within normal range. A plain roentgenogram of the chest and the abdomen showed grossly dilated large bowel loops, multiple air–fluid levels, absence of any free intraperitoneal air, and a crescent-shaped soft tissue density projecting into the gas of the large bowel ( Fig. 1 ). Ultrasound of the abdomen revealed an intraluminal echogenic content telescoping into distal lumen giving “target appearance” with mild proximal upstream dilatation. Hence, a provisional diagnosis of acute intestinal obstruction secondary to intussusception was made.

**Fig. 2:**
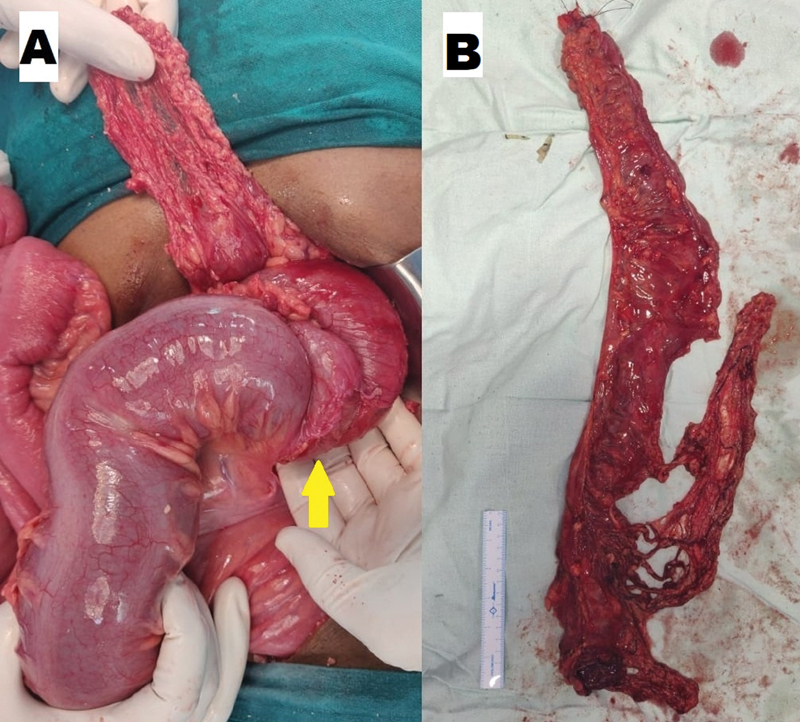
Intraoperative photograph depicting ( **A** ) constriction at splenic flexure with dilated adjacent distal colon and ( **B** ) resected specimen of the large bowel.

**Fig. 3:**
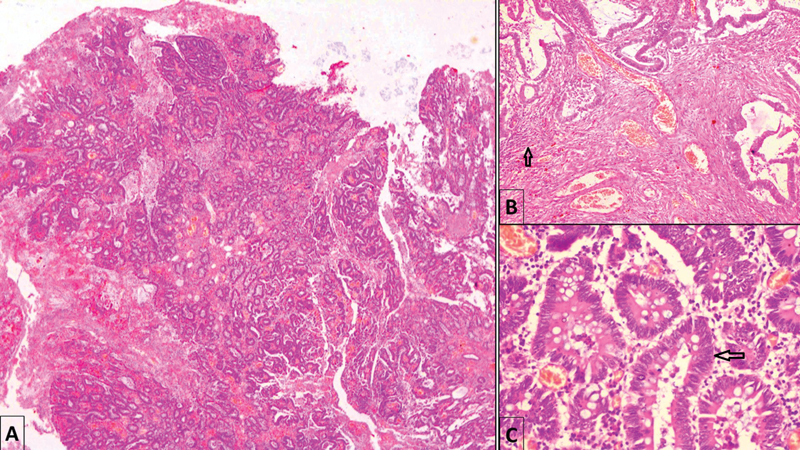
Histopathological examination after hematoxylin and eosin stain demonstrating ( **A** ) well-differentiated adenocarcinoma (×40), ( **B** ) tumor cells infiltrating into the muscularis propria (×100), and ( **C** ) tumor cells arranged in a glandular pattern with a moderate degree of nuclear pleomorphism (×400).

Initial resuscitation that preceded emergency surgery included oxygen supplementation, nasogastric decompression, intravenous fluids, and antibiotics. A note of dilated large bowel loops due to colocolic intussusception, present from distal transverse colon till sigmoid colon, was made on exploration of the abdomen. On gentle palpation, tight constriction at the splenic flexure due to telescoping of distal transverse colon and adjoining greater omentum into the descending colon could be appreciated. The healthy condition of the involved large bowel segment warranted a left hemicolectomy with resection from mid-transverse to the sigmoid colon followed by a primary end-to-end colocolic anastomosis ( Fig. 2 ). The patient was allowed orally on the fourth postoperative day on return of bowel function and went home with a healthy wound and no abdominal complaints. The resected mass sent for histopathological examination showed gray–white areas with hemorrhage. Finally, the microscopic report was suggestive of a well-differentiated adenocarcinoma (PT2N0M0) arising in a tubular adenoma polyp, with no lymph vascular invasion or perineural invasion ( Fig. 3 ). Hence, the final diagnosis was acute intestinal obstruction secondary to malignant left-sided colocolic intussusception.

## Discussion

Intussusception could be described as an “introversion” of the proximal bowel with its mesenteric fold within the lumen of the adjacent distal bowel as a result of overzealous or impaired peristalsis, further obstructing the free passage of intestinal contents and, more severely, compromising the mesenteric vascular flow of the intussuscepted segment. Adult intussusception is uncommon; it accounts for only 5% of all cases of intussusception. The clinical presentation of intussusception varies from acute, subacute, to chronic nonspecific symptoms. 3 Adults usually present with nonspecific and often long-standing complaints. The classic triad of abdominal mass, hemoglobin-positive stools, and tenderness of intussusception are rarely found in adults. 3 Literature states that only ∼1 to 5% of intestinal obstructions are due to intussusception. 4 Vomiting, gastrointestinal bleeding, and change in bowel habits are some of the nonspecific symptoms of intussusception that were the presenting complaints of our patient.

Intussusception can be broadly classified into enteric (jejunojejunal, ileoileal), ileocecal, ileocolic, and colonic based on the location. Ileocolic intussusception is the most common type, accounting for 80% of cases in children. Left-sided adult colon intussusception is a rare entity. 5 The patient, described herein, had colocolic intussusception (transverse colon prolapsing into the descending colon). Intussusception can also be classified according to the etiology (benign, malignant, and idiopathic). Small bowel intussusceptions are usually benign unlike large bowel, which are mostly malignant. A sessile polypoid mass measuring 6 × 5 cm was found, as the lead point at the level of mid-transverse colon, postresection in our patient.

Only 8 to 10% of adult intussusceptions are idiopathic, unlike in children which are 90%. In adult patients, intussusception lead points are typically pathological in 90% of cases, 65% of which are neoplastic in nature. 6 Colonic intussusception usually has a malignant pathology and needs high suspicion. Adenocarcinoma is the most common etiology of malignant colonic cases. In subjects presenting with enteric malignant intussusception, metastatic melanomas are the commonest. In benign causes, Meckel's diverticulum and lipoma rank first in enteric and colonic intussusception, respectively. 7 8

The diagnosis of intussusception is rarely made preoperatively. Abdominal radiographs, though not sensitive or specific, are the first diagnostic tool in emergency and may help in identifying the site of obstruction. An ultrasound of the abdomen may be less useful in adults, as often cannot identify the pathologic lead point but quite handy in the setting of a palpable abdominal mass. Barium scan, diagnostic and therapeutic modality in the children, has been replaced by an abdominal computed tomography (CT) scan in adults because it has been proved to be more informative and most sensitive. Stacked coin, coil-spring appearance, or cup-shaped filling defect is characteristically demonstrated in barium studies. 9 CT scan helps in revealing the site and cause of intussusception (underlying pathology) apart from the diagnosis itself. It has a diagnostic accuracy of 58 to 100%. Tomography may be helpful in judging the degree of vascular compromise if walls of the intussusceptum demonstrate any fluid or gas collection. 10 The presentation of acute intestinal obstruction with peritonism and visualization of “target sign” on ultrasonography led to the decision of upfront surgery in the described patient.

Most surgeons agree on the fact that laparotomy is required. The current treatment strategy is to go for resection without reduction to avoid the risk of seeding and dissemination of tumor cells. 11 On the contrary, various case reports have shown limited resection of bowel after reduction preoperatively. So, if a benign etiology is diagnosed preoperatively by colonoscopy, it is suggested that we can attempt the reduction of intussusception. Colonoscopy, a very efficient and safe method of treatment for intussusceptions in children, has limited therapeutic role in adults. 12 It has been reported that left-sided or rectosigmoid colon resection is performed with construction of proximal stoma specially in cases of emergency surgery. Our patient could be successfully managed with a primary anastomosis without stoma due to the absence of intraperitoneal contamination and the involved bowel segment being nongangrenous. The laparoscopic approach could be feasible in selected cases only because when a bowel obstruction occurs, bowel edema is developed, and little space is left in the abdominal cavity. 13

## Conclusion

Although rare, left-sided colocolic intussusception in adults requires the surgeon to understand the epidemiology and various treatment options. Preoperative radiology facilitates diagnosis. En bloc resection is the preferred surgical treatment for large bowel intussusception due to underlying pathologic lead point. In cases with benign etiology, unnecessary resections are avoided to prevent short bowel syndrome. A covering ileostomy may be avoided in healthy bowel or minimal peritoneal contamination.
